# Integrated Proteomics and Metabolomics Can Shed Light on Potential Biomarkers of Inflammation and Infection in Chicken Embryos as an Experimental Model

**DOI:** 10.1021/acs.jproteome.6c00153

**Published:** 2026-09-01

**Authors:** Simone Sommerfeld, Hebreia Oliveira Almeida Souza, Mario Machado Martins, Bianca Uliana Picolo, Isabelle Ezequiel Pedrosa, Bianca de Jesus e Silva, Anna Luiza Filsner Dias Strack, Danielle Silva Vieira, Lara Reis Gomes, Murilo Vieira da Silva, Victor Aguiar de Souza Penha, Belchiolina Beatriz Fonseca

**Affiliations:** † Graduate Program of Veterinary Science of Federal University of Uberlândia, Rua Ceará Bloco2D, Campus Umuarama—Bairro Umuarama, Uberlândia, MG CEP 38405-302, Brazil; ‡ Graduate Program of Genetics and Biochemistry of Federal University of Uberlândia Rua AcreBloco 2E, Campus Umuarama—Bairro Umuarama, Uberlândia, MG CEP 38400-902, Brazil; § Laboratory of Ticks and Other Apterous Arthropods, Oswaldo Cruz Institute, Oswaldo Cruz Foundation, Rio de Janeiro, Rio de Janeiro 21040-900, Brazil

**Keywords:** alternative model, protein, in ovo, 3R’s principle

## Abstract

*Salmonella* Pullorum (SP) infection has been proposed as an experimental model of inflammation in late-stage chicken embryos (CEs). We refined this model by inoculating 9.5 day embryos via allantois with a low SP dose and evaluating outcomes at 24 and 48 h postinfection. This approach aims to provide a framework for systematically exploring early molecular events triggered by infection and inflammation while using the CE as an ethically acceptable and biologically relevant model of systemic inflammation. Clinical parameters (mortality and weight), serum proteomics, and untargeted metabolomics were assessed. Proteomics was performed by LC–MS/MS, followed by PANTHER and KEGG functional enrichment; metabolomics was performed to assess serum alterations. Mortality increased at 48 h postinfection (30%), and surviving embryos showed a trend toward reduced weight. Proteomic profiling revealed an increase in overall protein concentrations in the infected group compared to the control, despite high zero inflation, with α-2-macroglobulin significantly upregulated at 48 h, indicating an endogenous immune response. For pathway-level enrichment, Bayesian analysis suggested subtle effects on proteome dynamics. Metabolomics identified time-dependent changes, including increased levels of α-tocopherol-related and phenylalanine-derived metabolites and decreased levels of oxidized fatty acid derivatives at 48 h. These findings support the CE model before 12 days as an ethically refined and sensitive system for studying acute inflammatory responses.

## Graphical abstract

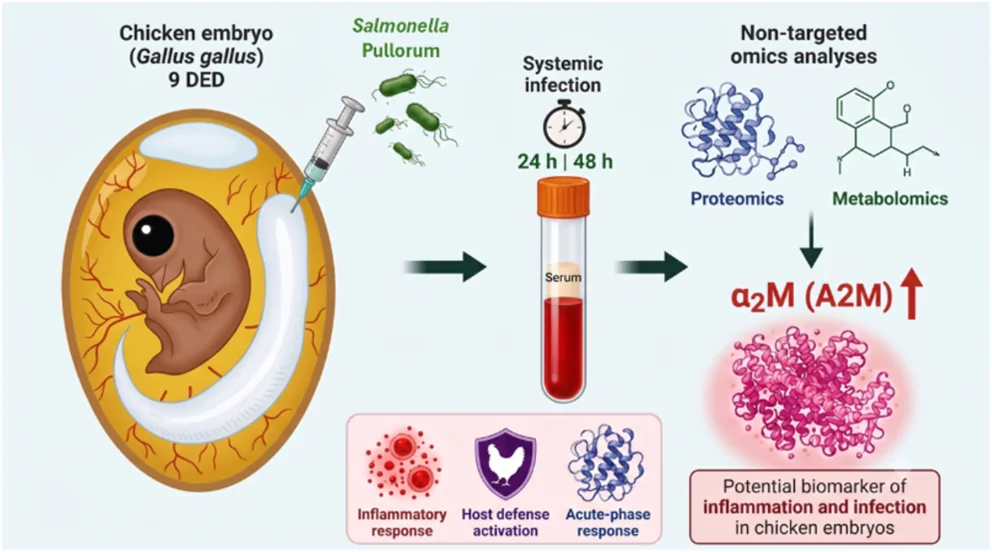

## Introduction

Understanding systemic responses to infection and inflammation, particularly in the context of identifying early molecular events triggered by pathogenic exposure, is a key goal of biomedical research. Traditionally, mammalian models have been used to investigate host responses.^1^ However, out of respect for animal welfare and in line with ethical research practices, scientists are increasingly turning to alternative models that follow the principles of replacement, reduction, and refinement (the 3Rs). In addition to its ethical advantages, the chicken embryo model is also biologically relevant to inflammation research, as several inflammatory pathways and acute-phase response mechanisms are evolutionarily conserved among vertebrates. In this context, the chicken embryo (CE) presents a promising experimental platform, particularly from a refinement perspective, as it allows the investigation of host–pathogen interactions during early developmental stages prior to the full establishment of pain perception.^2^

The CE offers several practical advantages, including low cost, high accessibility, and faster results.^3^ Importantly, embryos younger than 12 days of development (DED) are widely considered incapable of perceiving pain due to incomplete maturation of nociceptive circuits.^4^ As such, the CE before 12 DED represents a biologically relevant and ethically acceptable model for studying the systemic effects of infection and inflammation. Despite its increasing use in virology^5^ and toxicology,^6^ its application as an inflammation model by bacterial infection, particularly with a focus on systemic proteomic and metabolomic changes, remains limited.

Proteomic profiling enables the detection of subtle shifts in protein expression and functional activity, offering valuable insights into the molecular mechanisms underlying host responses.^7^ However, studying the proteome in embryonic tissues introduces analytical challenges, as limited functional annotation for *Gallus gallus* in current databases further complicates interpretation. Complementary metabolomic profiling can help address some of these challenges by capturing dynamic changes in small-molecule metabolites, providing a broader view of cellular responses, and revealing functional shifts in antioxidant, lipid, and amino acid pathways that may not be evident from proteomic data alone.^8^

Here, we investigated early systemic responses to *Salmonella* Pullorum (SP), as an experimental model of infection and inflammation, in the CE before 12 DED and how these responses evolve over time at 24- and 48 h postinfection. To address this, we combined nontarget proteomic and metabolomic profiling with Bayesian statistical modeling, multivariate analyses, and functional annotation to capture coordinated changes in proteins, metabolites, and related biological pathways. This approach provides a framework for systematically exploring early molecular events triggered by infection, using the CE as an ethically acceptable and biologically relevant model of systemic inflammation.

## Experimental Procedures

The experiments were conducted at the Poultry Incubation Laboratory (LIAVE), Laboratory of Infectious and Contagious Diseases (LADOC), and Nanobiotechnology Laboratory Prof. Luiz Ricardo Goulart Filho (NANOS) at the Federal University of Uberlândia (UFU). The project was evaluated and approved by the Animal Ethics Committee (CEUA) of UFU, under protocol numbers 008/21 and 23117.010881/2024-41. All procedures followed the standards and regulations established by the National Council for the Control of Animal Experimentation (CONCEA).

### Chicken Embryo Assays

We used SP as a model for infection and inflammation, based on our previous experience with this bacterium in CE at later developmental stages.^9^ Since we already knew it could cause complete embryo mortality at earlier stages, we used a much lower concentration of the pathogen (2 log CFU/mL). A total of 80 embryonated eggs from the Hy-Line strain of *Gallus gallus* were used and maintained in an automatic incubator (Premium Ecológica) at 37 °C and 55% humidity. For proteomic analysis, a minimum of 10 eggs per group were used at each time point. However, because metabolomic analysis required a larger sample volume, additional eggs were incubated to ensure sufficient samples. Thus, all viable embryos providing sufficient sample volume were included in the metabolomic analysis. This approach was adopted because metabolomic analyses generally require more samples for robust downstream evaluation. In Supporting Table 1, it is possible to observe the total number of samples tested. On day 9.5 DED, the CEs were divided into two groups: 40 CEs formed the positive control (PC), inoculated in the allatoic cavity with 100 μL of 2 log CFU/mL of SP, and 40 CEs formed the negative control (NC), inoculated with 100 μL of 0.85% saline solution. The embryonated eggs were weighed on the day of inoculation and monitored daily for mortality assessment using candling. At 10.5 DED and 11.5 DED (24 and 48 h postinfection, respectively), the mortality and gross damage were analyzed, the surviving embryos were weighed using an analytical balance (Bel LW303iH—0001 g × 310 g), and blood samples were collected from the umbilical vein from each group (PC and NC). After clot formation, blood was centrifuged at 2500*g* for 5 min to obtain the serum, which was transferred to sterile tubes and stored at −80 °C until further analysis.

### Proteomics Analysis

A total of ten samples of each group from embryos collected 24 and 48 h postinfection were used for proteomics analysis. For in-solution protein digestion, 50 μL of blood serum was treated for 5 min with 1% RapiGest SF (w/v) (Waters, Milford, MA) and 0.5 M dithiothreitol (DTT) in an ultrasonic bath. Samples were then incubated at 60 °C for 45 min under constant agitation, followed by alkylation with 0.5 M iodoacetamide (IAA) and incubation at 37 °C for 60 min in the dark. Proteins were digested with trypsin (20 ng/μL) at 37 °C overnight with continuous agitation. Digestion was stopped by acidification with 0.5% trifluoroacetic acid (TFA), followed by incubation at room temperature for 60 min. Precipitates were removed by centrifugation at 14,500 g for 10 min. The resulting supernatants were desalted, concentrated using ZipTips (Sigma-Aldrich) containing a C18 stationary phase, preconditioned, and washed with 0.1% TFA. Peptides were eluted using a 50:50 acetonitrile:water solution containing 0.1% TFA for subsequent proteomic analysis.

Mass spectrometry analysis was performed using a high-resolution quadrupole time-of-flight (Q-TOF) mass spectrometer (Agilent 6520B) coupled to a liquid chromatography system (Agilent Infinity 1260) equipped with an electrospray ionization (ESI) source. Peptide separation was achieved on an AdvanceBio Peptide Mapping column (Agilent Technologies) using a multistep acetonitrile gradient over 400 min at a constant flow rate of 0.4 mL/min. Ionization settings included a drying gas flow of 8 L/min at 325 °C, a nebulizer pressure set to 45 psi, and a capillary voltage of 4 kV. In the MS/MS methodology, collision energy varied according to peptide mass. For fragmentation, collision energy was selected using the formula ((Slope) × (*m*/*z*)/100 + Offset), with slope and offset values of 3 and 5, respectively. Data were acquired in positive ion mode and stored in centroid and profile formats over an *m*/*z* range of 55–1500. Proteins were identified using high-resolution mass measurements with a mass-accuracy threshold of <10 ppm and tandem MS/MS spectra. Data interpretation was conducted using the Spectrum Mill MS Proteomics Workbench (Agilent Technologies), with database searches restricted to*Gallus gallus*sequences from UniProt.

Mass spectrometry proteomics raw data were deposited in the ProteomeXchange Consortium via the PRIDE repository (PXD072137).

### Metabolomics Analysis

For metabolite extraction, 50 μL of serum was mixed with 1000 μL of spectroscopic-grade methanol, resulting in a 1:20 dilution. The samples were vortex-mixed for 5 min to ensure proper protein precipitation and metabolite extraction, then centrifuged at 15,000*g* for 15 min at room temperature. After centrifugation, the supernatant was carefully transferred to a clean microtube and concentrated under vacuum using a SpeedVac system overnight until complete evaporation of the solvent. The dried extracts were stored at −20 °C until further analysis. Prior to chromatographic analysis, samples were reconstituted in methanol, vortexed, and filtered through a 0.22 μm membrane filter.

Metabolite analyses were performed using high-performance liquid chromatography coupled to mass spectrometry (HPLC–MS) on an Agilent Infinity 1260 HPLC system coupled to an Agilent 6520 quadrupole time-of-flight (Q-TOF) mass spectrometer equipped with an electrospray ionization (ESI) source. Chromatographic separation was achieved using an Agilent Poroshell 120 column (2.1 mm × 50 mm, 2.7 μm). The mobile phase consisted of water acidified with 0.1% (v/v) formic acid (A) and methanol (B), using the following gradient: 10% B at 0 min, 98% B from 0 to 10 min, and 98% B from 10 to 17 min. The injection volume was 4 μL. The mass spectrometer was operated under the following conditions: a pressure of 20 psi, drying a gas flow of 8 L/min at 220 °C, and a capillary voltage of 4.5 kV.

Raw data were processed by using MassHunter Qualitative Analysis software (version 10.0). Molecular features were extracted using the molecular feature extractor (MFE) algorithm and converted to CEF format. Feature alignment, filtering, and statistical analyses were performed using Agilent Mass Profiler Professional (MPP) software (ver. B.13.1.1). Filtering criteria included a minimum absolute abundance of 5 000 counts, with all charge states allowed. Alignment parameters were set to a retention-time tolerance of 0.15 min and a mass-accuracy window of 15 ppm plus 2 mDa.

### Functional Enrichment Analysis

Functional enrichment analysis was conducted to explore the biological roles of all proteins identified in the proteomic data set. The Protein ANalysis THrough Evolutionary Relationships (PANTHER) classification system was used via its online web interface to annotate biological processes, molecular functions, and pathways,^10^ while the Kyoto Encyclopedia of Genes and Genomes (KEGG) database was used via its respective online platform exclusively for pathway analysis.^11^

### Multivariate and Differential Abundance Analyses for Omics

Omics data sets (proteomics and metabolomics) were analyzed using a unified multivariate and differential abundance framework, with adaptations according to the distributional properties of each data set. All analyses were conducted separately for 24 and 48 h postinfection to avoid confounding temporal effects.

#### Data Structure and Preprocessing

Proteomic and metabolomic data were structured as sample-by-feature matrices. For metabolomics, data originally provided in a long format were converted to a wide format prior to analysis.

Missing metabolomic values were interpreted as nondetected compounds and retained as missing during preprocessing. For distance-based multivariate analyses, missing values were imputed by using the median value of the corresponding metabolite to ensure numerical stability. Zero values were preserved as the true measurements.

Prior to multivariate analyses, metabolite intensities were mean-centered and variance-scaled. Proteomic concentration values were analyzed on their original scale, accounting for censoring as described below.

#### Multivariate Composition Analyses

To explore the overall variance structure and potential separation between experimental conditions (NC vs PC), principal component analysis (PCA) was performed using the prcomp­() function in R.

Distance-based analyses were conducted using both Euclidean and Bray–Curtis dissimilarities, followed by Principal Coordinates Analysis (PCoA) for visualization.

Group-level differences in multivariate omics composition were formally tested using PERMANOVA (Adonis2 function, vegan package) with 999 permutations. These analyses quantified the proportion of variance explained by the infection status and assessed whether observed group differences exceeded those expected by chance.

#### Differential Abundance Analyses

Feature-level differential abundance was evaluated using approaches tailored to the distributional characteristics of each data set.

Proteomic data exhibited strong zero inflation, suggesting potential left-censoring at zero. Therefore, Bayesian Tobit mixed-effects models were fitted using the brms package in R. Protein identity (“Compound”) was included as a random intercept to account for protein-specific variability, and infection status (“positive” or “negative”) was modeled as a fixed effect. Models assumed a Gaussian likelihood with lower censoring at zero. Four Markov chain Monte Carlo (MCMC) chains were run with 4000 iterations each (1000 warm-up) using adapt_delta = 0.95 and max_treedepth = 15. Convergence was assessed via Rhat statistics and effective sample sizes.

In contrast, metabolomic data showed minimal zero inflation and no substantial censoring. Therefore, conventional linear modeling approaches were deemed appropriate. Differential abundance testing was performed using the limma package, followed by empirical Bayes moderation (eBayes function). Adjusted p-values were calculated using the Benjamini–Hochberg false discovery rate (FDR) correction to account for multiple hypothesis testing across proteins and functional categories. Because the FDR procedure ranks all p-values simultaneously and applies a monotonicity constraint during adjustment, multiple features with similar raw p-values may converge to identical adjusted p-values after correction. This pattern is common in high-dimensional omics data sets, particularly when large numbers of comparisons are performed, and relatively few features exhibit strong statistical separation. For example, several proteins with raw p-values ranging from approximately 0.007 to 0.035 yielded identical adjusted p-values following FDR correction due to the cumulative ranking structure of the Benjamini–Hochberg procedure. All statistical analyses were conducted in R version 4.5.3 (2026-03-11) (R Core Team, 2026). Major packages included brms v2.23.0, vegan v2.7-1, limma v3.66.0, glmmTMB v1.1.12, tidyverse v2.0.0, FactoMineR v2.14, ggplot2 v4.0.0, lme4 v1.1-37, and data.table v1.17.8.

#### Functional Annotation Analyses (Proteomics)

For proteomic data, identified proteins were first annotated according to three Gene Ontology (GO) hierarchies: pathway, biological process, and molecular function. Functional annotations were assigned by mapping each protein to its associated GO terms using the corresponding annotation database. Separate sample-by-function matrices were then generated for each GO level. In these matrices, rows represented individual samples and columns represented functional categories, while cell entries corresponded to the cumulative normalized abundance of all proteins assigned to a given functional annotation within each sample. Because individual proteins may participate in multiple biological functions, proteins were allowed to contribute to more than one GO category. To reduce excessive sparsity and improve downstream model stability, functional categories with minimal representation across samples were removed prior to analysis. These sample-by-function matrices served as the basis for all subsequent functional analyses. Differences in functional enrichment between infection conditions were evaluated using Bayesian hierarchical linear models implemented in brms. In these models, infection status was included as a fixed effect, while functional annotations were incorporated as random intercepts to account for variability among GO categories. Models assumed Gaussian error distributions and used the same prior structure, sampling settings, and convergence diagnostics, as in the main analyses described above. To further evaluate overall functional compositional differences among samples, Bray–Curtis dissimilarity matrices were calculated from the sample-by-function matrices and used for ordination and multivariate analyses, including principal coordinates analysis (PCoA) and PERMANOVA with 999 permutations. Bray–Curtis dissimilarity was selected because functional abundance profiles were sparse and contained a high proportion of zeros, making a structure more comparable to compositional abundance data than to continuous multivariate data. Importantly, Bray–Curtis is relatively insensitive to joint absences and therefore better captures biologically meaningful differences in relative functional composition among samples. In parallel, principal component analysis (PCA) of centered and scaled functional abundance matrices was also performed to provide a complementary Euclidean-based ordination framework and facilitate visualization of dominant gradients in functional variation across infection conditions.^1^

## Results

### Chicken Embryo Weight and Mortality

There was no mortality in embryos after 24 h postinfection. After the collection of 20 embryos of each group, the remaining embryos were incubated for another 48 h before the new collection; mortality was then assessed by candling. In the PC group, 6 of 20 remaining embryos had died (Table 1).

**Table 1:** Embryo Mortality Following Bacterial Inoculation

development day on inoculation	treatment group	no. of embryos	mortality at 24 h	mortality at 48 h
9.5	Inoculated	40	0/40 (0%)	6/20^a^ (30%)
9.5	Negative control	40	0/40 (0%)	0/20^a^ (0%)

a Since then, twenty embryos were euthanized at 24 h.

Detailed embryo allocation and sample distribution for proteomic and metabolomic analyses are provided in Supporting Table S1.

Surviving embryos inoculated at 9.5 DED and euthanized 48 h later were weighed. Although no statistically significant difference was found, the *p*-value (0.0506) was very low, suggesting a trend toward significance (Figure 1).

**Figure 1:**
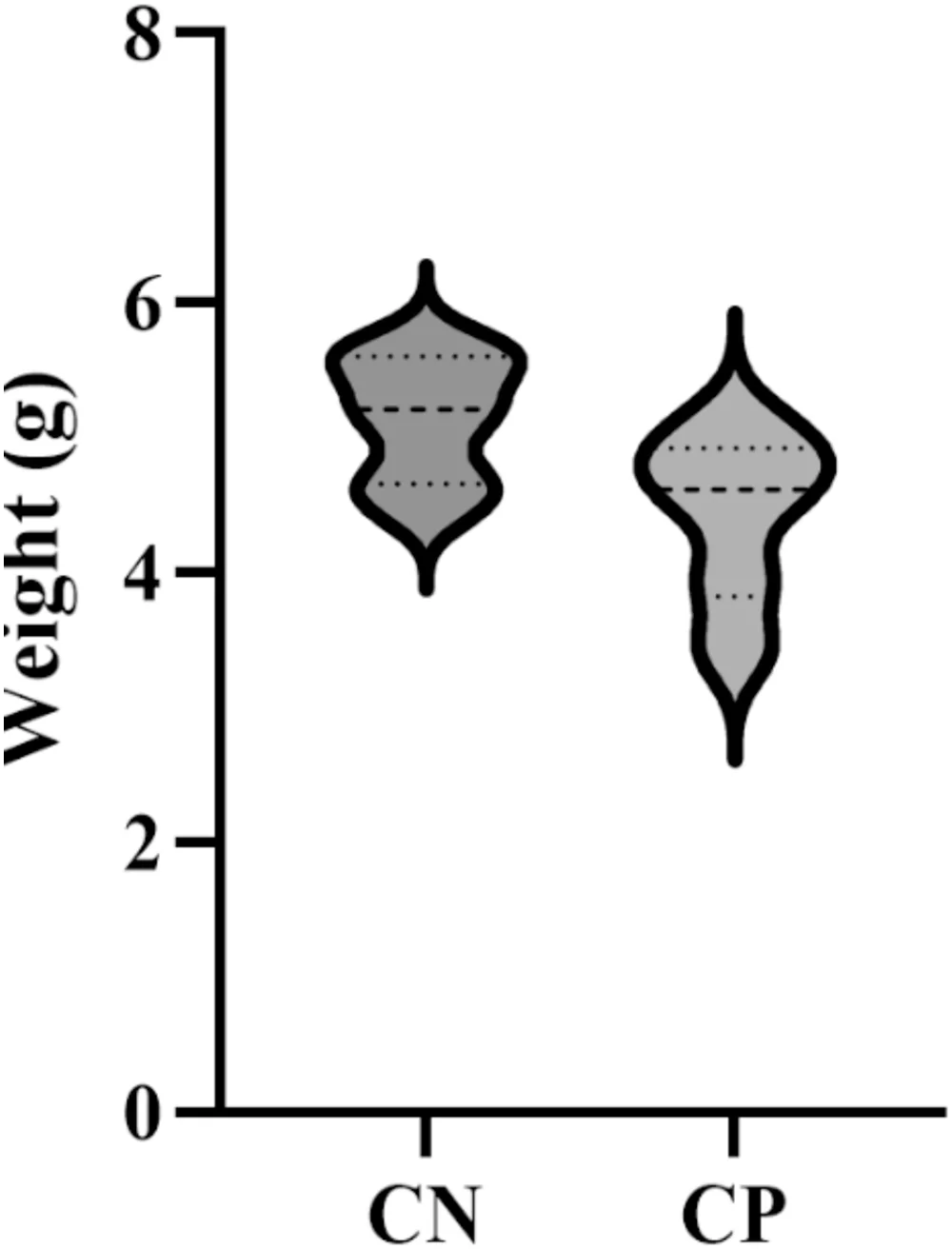
Weights of surviving embryos inoculated with saline (NC) or *Salmonella* Pullorum (PC) at 9.5 (B) embryonic days, evaluated 48 h postinfection. Although no statistically significant difference was found, the *p*-value (0.0506) was very low, suggesting a trend toward significance.

### Protein-Level Analysis

A total of 1164 proteins were identified in samples collected 24 h postinfection, and 1,463 proteins were identified at 48 h. Approximately 90% of the observations at both time points were zeros. This high proportion of zero values, consistent with patterns reported in untargeted proteomic data sets, is often attributed to signals falling below the detection limit.^12^

Only 5.76% and 56.6% of the proteins identified at 24 and 48 h postinfection, respectively, could be functionally classified using PANTHER and KEGG databases. This limitation is primarily due to the lack of experimental annotation available for *Gallus gallus* in the current reference databases. Consequently, our functional analysis was conducted solely on the subset of proteins with available annotations.

Results are presented separately for each time point to highlight the temporal dynamics in the host response.

### Twenty-Four Hours Postinfection

The Bayesian Tobit model estimated a small positive effect of the infected category on protein concentration (estimate = 0.20, 95% CI: [0.08, 0.31]; Figure 2), with a baseline concentration of 1.53 [1.38, 1.68] in the control group. We found ten proteins with large absolute log_2_ fold changes (positive or negative) and *p*-values of <0.05 in the comparison between the negative and positive groups. However, none of these proteins remained statistically significant after multiple testing correction (adjusted *p* > 0.48; Table 2). Model diagnostics were satisfactory (Rhat = 1.00), and substantial variation across proteins was observed (random intercept SD = 2.08; residual SD = 4.74).

**Figure 2:**
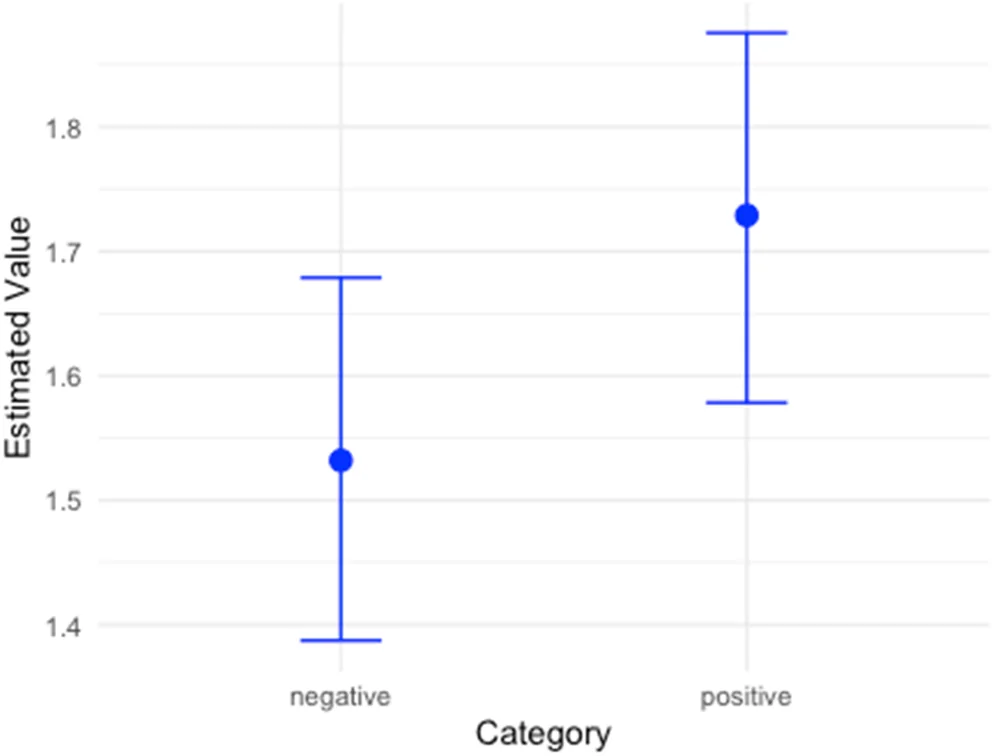
Estimated effect of infection status on overall protein concentration at 24 h post-treatment based on the Bayesian Tobit model. Points represent posterior mean estimates, and error bars represent 95% credible intervals for each infection category. The model accounted for left-censoring of protein concentration values and estimated posterior log-protein concentrations across groups. The positive (infected) group exhibited higher estimated protein concentrations than the negative (control) group. The *Y*-axis represents posterior mean estimated log-protein concentration derived from the Bayesian model.

**Table 2:** Top Ten Proteins with the Largest Absolute Log Fold Change and Corresponding Significance Values^a^

protein	logFC	*p*-value	adj.*p*-value
PocR ligand-binding domain-containing protein	–7.36218	0.007443	0.481581
DNA-directed RNA polymerase subunit β′	–5.88011	0.010626	0.481581
C40 family peptidase (Fragment)	–7.11622	0.023203	0.481581
Hemoglobin subunit α-D	5.981186	0.030754	0.481581
Large ribosomal subunit protein bL27	5.464271	0.030937	0.481581
Winged helix-turn-helix domain-containing protein	–6.161	0.031186	0.481581
Rne/Rng family ribonuclease (Fragment)	–5.09971	0.033392	0.481581
Amidohydrolase family protein (Fragment)	–5.0408	0.033854	0.481581
C4-type zinc ribbon domain-containing protein	–4.99478	0.03431	0.481581
DNA-protecting protein DprA	4.929941	0.034848	0.481581

a Negative values of logFC indicate lower expression in the infected group compared to controls, while positive values indicate higher expression. logFC: log fold change; *p*-values: unadjusted *p*-values; adj.*p*-value: adjusted *p*-values (FDR). No proteins reached statistical significance after the FDR correction.

For functional pathway analysis (*n* = 38), the Bayesian linear mixed-effects model estimated a modest effect of infection (estimate = 0.36, 95% CI: [−0.09, 0.79]), although the credible interval included zero (Table 3). Pathway-level variability was high (SD = 6.16), as was residual variance (SD = 5.77). Although several pathways exhibited notable fold changes, none met the criteria for statistical significance after FDR adjustment (Table 3). Compositional analyses (PERMANOVA: *R* ^2^ = 0.055, *F* = 1.05, *p* = 0.392; PCA and PCoA) revealed substantial overlap between infected and control samples.

**Table 3:** Top Ten Functional Pathways Ranked by Evidence of Differential Abundance^a^

pathway	logFC	*p-*value	adj. *p*-value
huntington_disease_p00029	–3.400735	0.05434367	0.5358081
vitamin_d_metabolism_and_pathway_p04396	5.001452	0.12413911	0.5358081
egf_receptor_signaling_pathway_p00018	2.782077	0.1437023	0.5358081
fgf_signaling_pathway_p00021	2.782077	0.1437023	0.5358081
parkinson_disease_p00049	2.782077	0.1437023	0.5358081
cadherin_signaling_pathway_p00012	–2.32676	0.19241542	0.5358081
wnt_signaling_pathway_p00057	–2.32676	0.19241542	0.5358081
vitamin_digestion_and_absorption	3.144938	0.2008284	0.5358081
influenza_a	4.051805	0.20700077	0.5358081
neuroactive_ligand_receptor_interaction	4.051805	0.20700077	0.5358081

a Negative values of logFC indicate lower expression in the infected group compared to controls, while positive values indicate higher expression. logFC: log fold change; *p*-values: unadjusted *p*-values; adj. *p*-value: adjusted *p*-values (FDR). No pathways reached statistical significance after FDR correction.

In the analysis of biological processes (*n* = 9), a slightly stronger effect was detected (estimate = 0.60, 95% CI: [0.17, 1.03]). Among the processes, the *developmental process* showed a nominally significant unadjusted *p*-value (*p* < 0.05); however, this result did not remain significant after correction for multiple comparisons. Overall, none of the biological processes met the significance threshold following FDR adjustment (Table 4). The intercept for the control group was estimated at 5.92 [1.72, 9.89], with high residual variability (SD = 6.83) and random-effect variability (SD = 6.53). PERMANOVA showed no significant group separation (*R* ^2^ = 0.073, *F* = 1.41, *p* = 0.36), and ordination analyses confirmed a strong group overlap.

**Table 4:** Differential Abundance Results for Biological Processes^a^

biological process	logFC	*p*-value	adj. *p*-value
developmental_process	–1.27621554	0.03595366	0.3595366
localization	1.94344828	0.09656887	0.4828444
homeostatic_process	3.14072237	0.17732355	0.5330334
metabolic_process	1.60190989	0.21321337	0.5330334
multicellular_organismal_process	–0.69732012	0.38378537	0.7675707
cellular_process	0.36860625	0.62442156	0.8604248
interspecies_interaction	0.3022397	0.6883398	0.8604248
response_to_stimulus	0.15187429	0.83933781	0.9325976
biological_regulation	0.02177744	0.96911855	0.9691185

a Negative values of logFC indicate lower expression in the infected group compared to controls, while positive values indicate higher expression. logFC: log fold change; *p*-values: unadjusted p-values; adj. *p*-value: adjusted *p*-values (FDR). No biological process reached statistical significance after the FDR correction.

Analysis of molecular functions (*n* = 9) showed a similar result (estimate = 0.56, 95% CI: [0.15, 1.00]; control intercept = 4.26 [1.63, 6.82]; residual SD = 6.50; random-effects SD = 4.13). As in previous levels, multivariate compositional analysis did not indicate significant differences (PERMANOVA: *R* ^2^ = 0.069, *F* = 1.33, *p* = 0.22), and none of the functions passed the FDR threshold for statistical significance (Table 5), with ordination analyses confirming strong group overlap.

**Table 5:** Differential Abundance Results for Molecular Functions^a^

molecular function	logFC	*p*-value	adj. *p*-value
molecular_adaptor_activity	–1.832773	0.107470	0.639810
transporter_activity	2.404668	0.129623	0.639810
structural_molecule_activity	–1.316140	0.198367	0.639810
binding	0.972101	0.269256	0.639810
catalytic_activity	1.205032	0.319905	0.639810
antioxidant_activity	1.473245	0.473227	0.738612
atp_dependent_activity	1.675976	0.517028	0.738612
transcription_regulator_activity	0.109857	0.900841	0.971577
molecular_function_regulator_activity	0.051439	0.971577	0.971577

a Negative values of logFC indicate lower expression in the infected group compared to controls, while positive values indicate higher expression. logFC: log fold change; *p*-values: unadjusted *p*-values; adj. *p*-value: adjusted *p*-values (FDR). No molecular function reached statistical significance after the FDR correction.

### Forty-Eight Hours Postinfection

As observed at 24 h, protein expression at 48 h postinfection remained highly zero-inflated (∼89.5%). The Bayesian Tobit model estimated a modest increase in overall protein concentrations in the infected group relative to controls (estimate = 0.26, 95% CI: [0.12, 0.40]), with a control-group intercept of 1.84 [1.70, 1.98]. This general effect suggests a mild but consistent shift in proteomic expression following infection. The overall distribution of posterior estimates is visualized in the credible interval plot (Figure 3). Despite the modest average change, ten proteins presented *p* < 0.05 (Table 6), but only one protein (α-2-macroglobulin-like protein 1) stood out with a highly significant and biologically meaningful increase. This protein exhibited a log_2_ fold change of 13.13 and remained significant after FDR correction (adjusted *p* = 0.0081), representing an over 8000-fold elevation in the infected group (Table 6).

**Figure 3:**
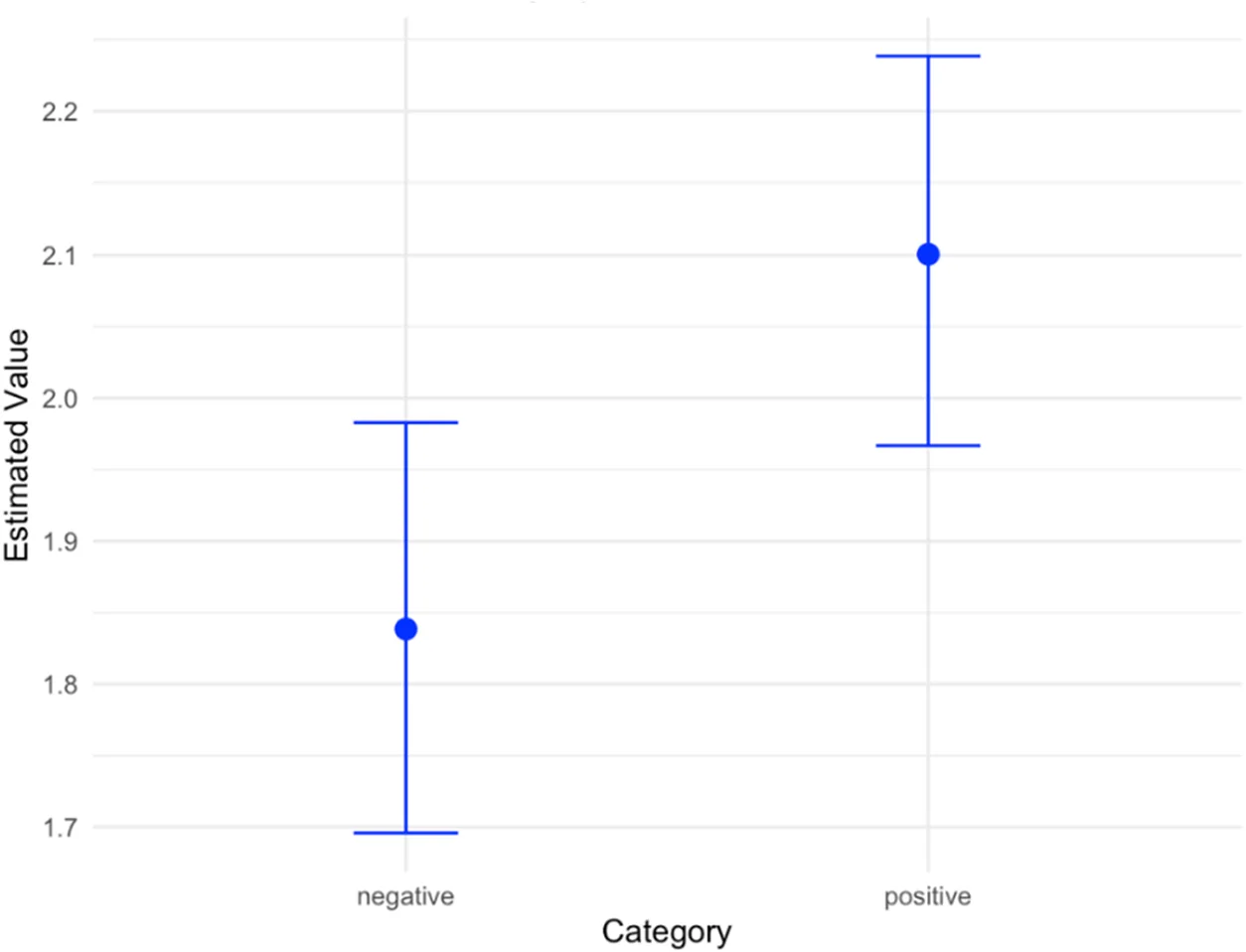
Estimated effect of infection status on overall protein concentration at 48 h post-treatment based on the Bayesian Tobit model. Points represent posterior mean estimates, and error bars represent 95% credible intervals for each infection category. The model accounted for left-censoring of protein concentration values and estimated posterior log-protein-concentration across groups. The positive (infected) group exhibited higher estimated protein concentrations than the negative (control) group. The *Y*-axis represents posterior mean estimated log-protein concentration derived from the Bayesian model.

**Table 6:** Top Ten Proteins Ranked by Evidence of Differential Abundance at 48 h Postinfection^a^

protein	logFC	*p*-value	adj. *p*-value
alpha_2_macroglobulin_like_protein_1	13.1272	552 × 10^–06^	0.0080
eh_domain_binding_protein_1_like_protein_1	9.5960	0.00281	0.5453
cadherin_domain_containing_protein	–7.7774	0.00315	0.5453
pit54_protein	8.2560	0.00978	0.5453
Plasminogen	7.1136	0.01245	0.5453
leucine_zipper_protein_1	–7.0448	0.01386	0.5453
fibrinogen_c_domain_containing_1	8.07286	0.01486	0.5453
rous_sarcoma_virus_transcription_enhancer_factor_iii	7.57050	0.01609	0.5453
protein_tyrosine_phosphatase	7.41027	0.01625	0.5453
ciliogenesis_and_planar_polarity_effector_complex_subunit_1	–6.4227	0.01629	0.5453

a Negative values of logFC indicate lower expression in the infected group compared to controls, while positive values indicate higher expression. logFC: log fold change; *p*-values: unadjusted p-values; adj. *p*-value: adjusted *p*-values (FDR).

At the pathway level, the Bayesian hierarchical model estimated a significant increase in expression values in the infected group (estimate = 0.31, 95% CI: [0.12, 0.51]), indicating consistently higher pathway-associated concentrations compared with controls. The model accounted for moderate variability across pathways (random-effects SD = 3.28; residual SD = 5.23), with a control-group intercept of 3.13 [1.54, 4.70]. Despite these differences in magnitude, multivariate analysis did not detect significant compositional shifts (PERMANOVA: *F* = 1.14, *R* ^2^ = 0.066, *p* = 0.284), and ordination confirmed overlapping group structures. Several pathways exhibited relatively large log_2_ fold changes, and six pathways showed nominal significance (unadjusted *p* < 0.05). However, none of these results remained statistically significant after FDR adjustment (Table 7).

**Table 7:** Top Molecular Pathways Ranked by Evidence of Differential Abundance at 48 h Postinfection^a^

pathway	logFC	*p*-value	adj. *p*-value
plasminogen_activating_cascade_p00050	5.865	0.008789	0.65879
mitophagy_animal	–6.065	0.019005	0.65879
carbon_metabolism_gga01200	6.843	0.024512	0.65879
pyruvate_metabolism_gga00620	6.843	0.024512	0.65879
blood_coagulation_p00011	3.136	0.030551	0.65879
ppar_signaling_pathway	4.905	0.033103	0.65879
pi3_kinase_pathway_p00048	2.763	0.051977	0.65879
hormone_signaling_gga04081	2.701	0.055584	0.65879
transcription_regulation_by_bzip_transcription_factor_p00055	–3.344	0.059852	0.65879
angiotensin_ii_stimulated_signaling_through_g_proteins_and_beta_arrestin_p05911	2.759	0.066855	0.65879

a Negative values of logFC indicate lower expression in the infected group compared to controls, while positive values indicate higher expression. logFC: log fold change; *p*-values: unadjusted *p*-values; adj. *p*-value: adjusted *p*-values (FDR). No pathways reached statistical significance after the FDR correction.

At the biological process level (*n* = 18), the Bayesian hierarchical model estimated a small but statistically credible increase in the infected group (estimate = 0.21, 95% CI: [0.08, 0.33]), indicating slightly higher biological process-associated concentrations compared to controls. The model accounted for moderate variability across processes (random-effects SD = 3.28). Despite this shift in average abundance, multivariate compositional differences were not significant (PERMANOVA: *F* = 0.24, *R* ^2^ = 0.015, *p* = 0.89), and ordination confirmed strong overlap between groups. Differential abundance analysis revealed moderate log_2_ fold changes in some biological processes; however, none reached statistical significance either before or after adjustment for multiple comparisons (Table 8).

**Table 8:** Top Biological Processes Ranked by Evidence of Differential Abundance at 48 h Postinfection^a^

biological process	logFC	*p*-value	adj. *p*-value
rhythmic_process_go_0048511	1.5904	0.1816	0.9068
cellular_process_go_0009987	1.9694	0.2364	0.9068
multicellular_organismal_process_go_0032501	0.6508	0.2927	0.9068
locomotion_go_0040011	–0.9851	0.3234	0.9068
developmental_process	0.3288	0.5572	0.9068
biological_process_involved_in_interspecies_interaction_between_organisms_go_0044419	0.5457	0.5831	0.9068
metabolic_process_go_0008152	0.2566	0.6115	0.9068
homeostatic_process_go_0042592	0.3278	0.6793	0.9068
localization_go_0051179	0.1862	0.7008	0.9068
response_to_stimulus_go_0050896	0.1768	0.7541	0.9068

a Negative values of logFC indicate lower expression in the infected group compared to controls, while positive values indicate higher expression. logFC: log fold change; *p*-values: unadjusted *p*-values; adj. *p*-value: adjusted *p*-values (FDR). No biological process reached statistical significance after FDR correction.

At the molecular function level (*n* = 14), the Bayesian hierarchical model estimated a small but statistically credible increase in the infected group (estimate = 0.25, 95% CI: [0.08, 0.42]), suggesting slightly higher molecular function-related values compared to controls. The model accounted for moderate variability across functions (random-effects SD = 1.04). Despite this shift in average concentration, multivariate analysis did not indicate significant multivariate compositional differences between groups (PERMANOVA: *F* = 0.99, *R* ^2^ = 0.059, *p* = 0.42), and ordination confirmed substantial overlap. Differential abundance testing highlighted some functions with relatively high log_2_ fold changes, but none were statistically significant either before or after FDR correction (Table 9).

**Table 9:** Top Molecular Functions Ranked by Evidence of Differential Abundance at 48 h Postinfection^a^

molecular function	logFC	*p*-value	adj. *p*-value
antioxidant_activity_go_0016209	3.61626	0.13907	0.68963
transcription_regulator_activity_go_0140110	0.64910	0.21559	0.68963
structural_molecule_activity_go_0005198	0.83609	0.21573	0.68963
translation_regulator_activity_go_0045182	2.05640	0.26655	0.68963
molecular_function_regulator_activity_go_0098772	0.43516	0.29336	0.68963
cargo_receptor_activity_go_0038024	1.90976	0.35872	0.68963
atp_dependent_activity_go_0140657	–0.66552	0.39407	0.68963
catalytic_activity_go_0003824	0.34276	0.51208	0.79658
molecular_transducer_activity_go_0060089	–0.37933	0.56914	0.79680

a Negative values of logFC indicate lower expression in the infected group compared to controls, while positive values indicate higher expression. logFC: log fold change; *p*-values: unadjusted *p*-values; adj. *p*-value: adjusted *p*-values (FDR). No molecular function reached statistical significance after FDR correction.

### Metabolite-Level Analysis

Multivariate analyses revealed clear and statistically significant differences in metabolomic profiles between NC and PC conditions at both incubation times. At 24 h postinfection, principal component analysis (PCA) showed a visible separation between NC and PC samples along the first principal component, indicating distinct metabolomic signatures between treatments (Figure 4A). This pattern was corroborated by distance-based ordination using principal coordinates analysis (PCoA) of Bray–Curtis dissimilarities, which also showed partial clustering by experimental condition (Figure 4B).

**Figure 4:**
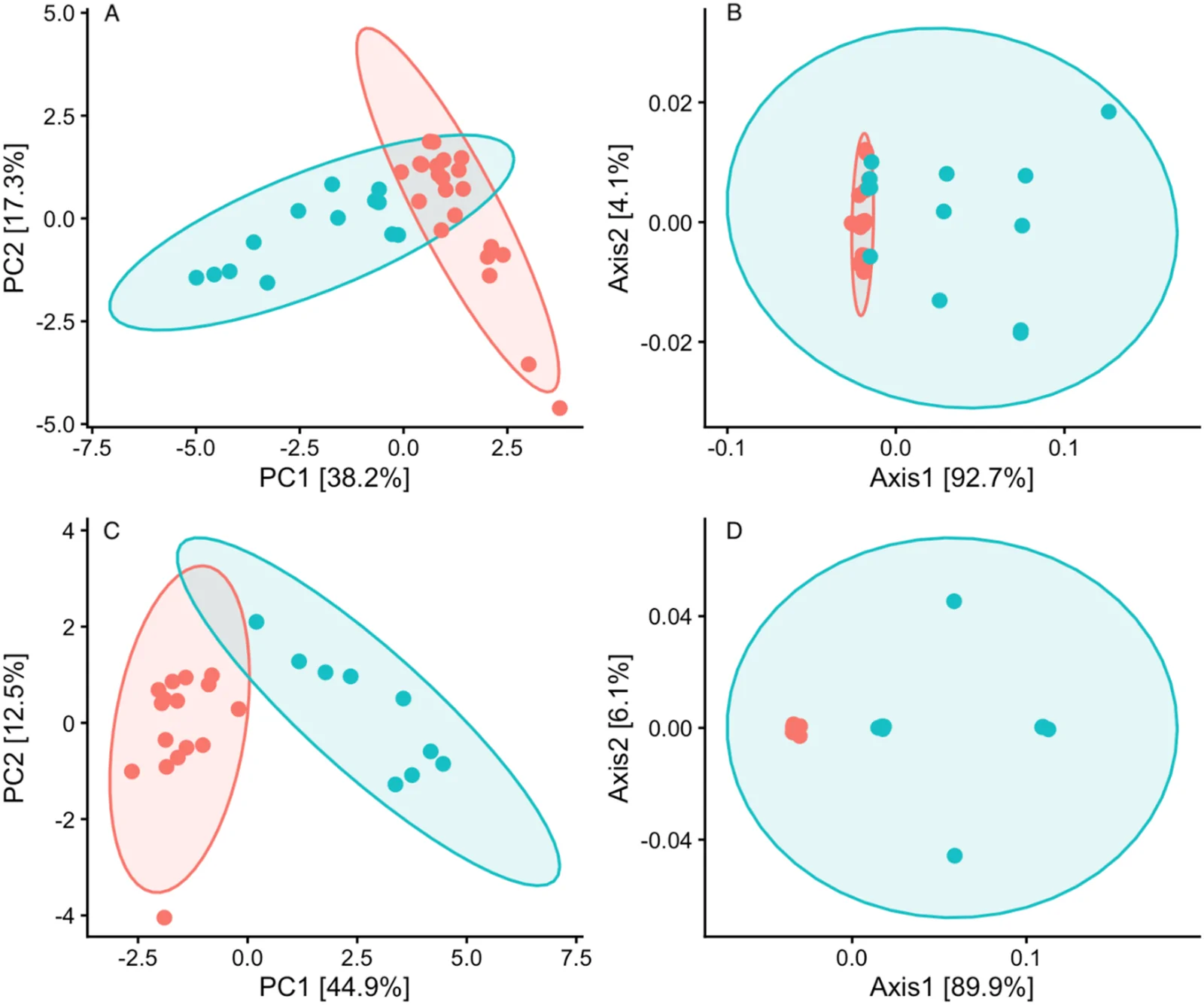
Multivariate analysis of metabolite profiles in chicken embryos at 24 and 48 h postinfection. Principal component analysis (PCA; A, C) and principal coordinates analysis (PCoA) of Bray–Curtis dissimilarities (B, D) were performed using autoscaled metabolite abundance data. Percent variance explained by each axis is indicated in parentheses. Shaded areas represent 95% confidence ellipses for each experimental group. NC samples are shown in orange, and PC samples are shown in green. Greater separation between groups was observed at 48 h postinfection, indicating increased metabolic divergence over time. The linear distribution observed in panel D likely reflects the use of Bray–Curtis dissimilarity on a relatively small metabolite feature set.

PERMANOVA confirmed that metabolomic composition differed significantly between NC and PC at 48 h postinfection (*F* = 11.84, *R* ^2^ = 0.264, *p* = 0.001), with treatment explaining approximately 26.4% of the total variance in metabolite profiles. At 48 h postinfection, the separation between NC and PC samples was even more pronounced. PCA revealed stronger divergence between groups along PC1, with reduced overlap compared to 24 h postinfection (Figure 4C). PCoA similarly showed clear separation between conditions in the ordination space (Figure 4D). Consistent with these visual patterns, PERMANOVA indicated a significant treatment effect at 48 h postinfection (*F* = 14.17, *R* ^2^ = 0.381, *p* = 0.001), with experimental condition explaining 38.1% of the total variance. This increase in explained variance relative to 24 h postinfection suggests that metabolomic differentiation between NC and PC intensifies over time. Across both time points, analyses were based on 12 metabolites, ensuring a direct comparability between days.

At 24 h postinfection, untargeted serum metabolomic analysis identified a limited number of metabolites that differed between PC and NC embryos. After adjustment for multiple comparisons, few metabolites remained statistically significant. Among the differentially detected compounds, changes were primarily characterized by decreased abundance of specific lipid-related metabolites, including 2R-hydroxylauric acid, which showed a statistically significant reduction and a notable fold change. Additional metabolites showed nominal differences between groups but were not significant after *p*-value adjustment. Features lacking high-confidence structural confirmation (MSI levels 1 and 2) were classified as unknown metabolites. These are reported for completeness but were not used for biological pathway interpretation (Table 10).

At 48 h postinfection, a distinct set of metabolites was differentially detected between infected and control embryos. Several metabolites remained statistically significant after *p*-value adjustment and displayed higher fold-change values. Increased abundance was observed for metabolites related to aromatic amino acid metabolism, including phenylacetaldehyde, which showed one of the highest fold-change values at this time point. In contrast, reduced levels of oxidized fatty acid derivatives, such as 16-hydroxy-10-oxohexadecanoic acid, were consistently detected in infected embryos. Additional metabolites showed significant differences but with lower fold-change values Table[Table tbl11].

**Table 10:** Top Ten Compounds Ranked by Evidence of Differential Abundance at 24 h Postinfection^a^

compounds	logFC	*p*-value	adj. *p*.value
α-CEHC	0.6917	8.11 × 10^–11^	0.0000
2R-hydroxylauric acid	–0.5558	4.92 × 10^–7^	0.0000
Unknown metabolite	–0.2115	2.27 × 10^–4^	0.0009
16-Hydroxy-10-oxohexadecanoic acid	–0.2029	6.72 × 10^–4^	0.0020
Unknown metabolite	–11.3574	8.98 × 10^–4^	0.0022
2-(4-Methyl-5-thiazolyl)ethyl butanoate	0.2833	5.07 × 10^–3^	0.0101
Unknown metabolite	–7.0088	1.70 × 10^–02^	0.0291
α-tocopheronolactone	0.3910	4.02 × 10^–02^	0.0603
3-Hydroxycoumarin	0.0230	1.42 × 10^–01^	0.1894
Emmotin A	–1.5987	3.13 × 10^–01^	0.3758

a Negative values of logFC indicate lower expression in PC compared to NC, while positive values indicate higher expression. logFC: log fold change; *p*-values: unadjusted *p*-values; adj. *p*.value: adjusted *p*-values (FDR).

**Table 11:** Top Ten Compounds Ranked by Evidence of Differential Abundance at 48 h Postinfection^a^

compounds	logFC	*p*-value	adj.*p*.value
Phenylacetaldehyde	17.9779	9.53 × 10^–20^	0.0000
Unknown metabolite	–0.3823	7.99 × 10^–10^	0.0000
α-CEHC	0.4501	1.77 × 10^–06^	0.0000
16-Hydroxy-10-oxohexadecanoic acid	–0.1180	6.93 × 10^–05^	0.0002
Unknown metabolite	0.0634	1.64 × 10^–04^	0.0004
Unknown metabolite	–8.2029	3.64 × 10^–02^	0.0547
Unknown metabolite	–8.0659	3.42 × 10^–02^	0.0547
Emmotin A	–0.6064	3.45 × 10^–02^	0.0547
alhpa-tocopheronolactone	0.5426	5.19 × 10^–02^	0.0692
Unknown metabolite	0.1436	2.01× 10^–01^	0.2406

a Negative values of logFC indicate lower expression in PC compared to NC, while positive values indicate higher expression. logFC: log fold change; *p*-values: unadjusted *p*-values; adj. *p*.value: adjusted *p*-values (FDR).

## Discussion

*Salmonella* Pullorum (SP) is a highly virulent embryonic pathogen that causes high mortality in CE. In our previous studies, we observed that the older the embryos, the more resistant they became to infection, with the degree of resistance depending on the bacterial dose.^9^ Therefore, in this study, we inoculated a very low dose (1 log CFU/CE) in an attempt to evaluate the surviving embryos. In this model, we observed an embryonic mortality rate of 30% after 48 h, similar to findings in older embryos exposed to higher doses and evaluated over the same period. This high mortality confirms that the bacteria were active and suggests that some embryos may have mounted an immune response to limit bacterial proliferation.^13^

This hypothesis is supported by our weight data: embryos in the PC group had lower body weights than controls, with a *p*-value close to significance (*p* = 0.0506), indicating a trend. Similarly, embryos inoculated with SP at 13 days of embryonic development (DED) and evaluated at 17 DED also exhibited weight loss.^9^ These findings support our hypothesis that despite their young age, some embryos initiate an immune response to counteract the pathogenicity of SP. To further explore the molecular mechanisms involved in this response, we performed a nontargeted proteomic analysis.

Nontargeted proteomics is a powerful approach to explore the biological responses and molecular pathways activated during infection. While we acknowledge the limitations of the incomplete protein database for our target species (*Gallus gallus*), only 5.76% and 56.6% of the identified proteins were functionally annotated at 24 and 48 h, respectively. Nevertheless, we emphasize that the proteomic results obtained were highly reliable.

Our findings are supported by robust statistical analyses that remain valid despite the inherent challenges of zero-inflated data. Therefore, although we recognize that some pathways, biological processes, and molecular functions may not have been detected or discussed due to database limitations, we are confident in the accuracy and biological relevance of the proteins and pathways identified.

Many of the values obtained for proteins, metabolic pathways, biological processes, and molecular mechanisms in our comparison between the two groups (PC and NC) were statistically significant at the unadjusted level (*p* < 0.05). However, these differences did not remain significant after correction for multiple testing. Despite this, we chose to discuss these findings as exploratory trends, in line with the approach supported by O’Connell et al.,^14^ who emphasize that in proteomics analyses with missing values, strict *p*-value adjustment can mask biologically meaningful signals and increase false negatives. Additionally, Rubin^15^ argued that in simple comparisons involving only two groups, adjusting *p*-values may not be necessary and can unnecessarily reduce statistical power. These perspectives suggest that, particularly in exploratory studies, consistent trends supported by biological plausibility and fold-change directionality may provide valuable insights, even in the absence of adjusted statistical significance.^16^

Although only one protein reached statistical significance after multiple testing correction, our results consistently revealed small but credible increases in the protein abundance and functional activity in infected samples. These effects were detected through Bayesian modeling under zero-inflated conditions, a common challenge in proteomic data sets.^12^

At 24 h postinfection, the Bayesian Tobit model indicated a modest increase in overall protein concentration in the infected group. Although no individual proteins met the significance threshold after FDR adjustment, the general upward trend may indicate subtle early proteomic responses to infection, although these effects remained weak relative to overall variability in the data set. For example, hemoglobin subunit α-D, known to function as a mitochondrial antioxidant in nonerythroid cells,^17^ was upregulated in positive samples, suggesting early oxidative stress responses in infected embryos.

The protein annotated as “Winged helix-turn-helix domain-containing protein” showed a tendency toward reduced abundance in infected embryos. It was in line with the only biological process with a negative fold change and significant unadjusted *p*-value: “developmental process.” This alignment suggests that infection may interfere with key regulatory mechanisms active during early embryogenesis. Biological processes were classified using the PANTHER system, which incorporates Gene Ontology data,^18^ and describes how living systems evolve over time. At early developmental stages, proper genome activation and transcription factor activity are crucial. Supporting this, Liao et al.^19^ reported dynamic expression of key transcription factor families during chicken embryogenesis, including Fox, belonging to the winged helix-turn-helix class, reinforcing the relevance of this protein’s downregulation within the broader context of developmental regulation.

The minimal changes observed in inflammatory pathways, evidenced by only one inflammation-related protein showing an unadjusted *p*-value <0.05, combined with the downregulation of development-associated proteins, align with the outright absence of mortality at 24 h postinfection. Considering that even highly virulent *Salmonella* strains typically cause significant embryo mortality only after ∼48 h.^13^

By 48 h postinfection, the expression pattern remained highly zero-inflated, yet the overall shift in protein abundance became more pronounced. The Bayesian Tobit model estimated a credible increase in protein levels in the infected group (estimate = 0.26, 95% CI: [0.12, 0.40]), reflecting a modest average increase in protein abundance in infected embryos. While most individual proteins did not reach statistical significance after multiple testing correction, α-2-macroglobulin-like protein 1 was significantly upregulated, indicating a targeted and biologically relevant response. This suggests that, alongside a subtle and diffuse modulation across many proteins, infection can also induce marked changes in selected components, which may reflect early activation of immune or stress pathways in the developing embryo.^20^

Although α2-macroglobulin (A2M) is known to be a ubiquitous plasma protein across vertebrates,^21,22^ its immunomodulatory role has been thoroughly documented only in mammalian species.^20,23,24^ In contrast, avian studies have focused almost exclusively on A2M (referred to as ovostatin in birds) in egg white, where it is synthesized by the oviduct and acts as an antibacterial, protease-inhibiting defense factor for the embryo.^25–27^

Importantly, no studies to date have examined A2M in chicken blood or liver in response to infection, and its presence in circulating avian tissues has remained largely unexplored. Intriguingly, however, multiple proteomic analyses of embryonic chicken liver, originally aimed at metabolic profiling,^28–30^ consistently report the presence of A2M isoforms in Supporting Data. In the present study, A2M abundance increased markedly in infected embryos, with a substantially higher fold change than in controls, particularly at 48 h postinfection. This pronounced upregulation, coinciding with the onset of embryo mortality, strongly indicates that A2M is not merely a passive residual component from maternal deposition but is dynamically regulated in response to infection. The magnitude and temporal pattern of this increase support its involvement in the embryonic inflammatory response and suggest that A2M may serve as a biomarker of inflammation and disease progression in developing embryos.

In mammals, A2M is a well-established acute-phase protein induced primarily by innate immune signals such as IL-6 and TNF-α, and functions as a broad-spectrum protease inhibitor, cytokine carrier, and modulator of immune cell activity, particularly macrophages and neutrophils. Besides, its immunomodulatory properties extend to adaptive immune processes, making it a key player in both arms of immunity.^20^ Although adaptive immunity is not fully functional at this embryonic stage, the observed increase in A2M may reflect innate immune activation in response to systemic infection. Its emergence only at 48 h, and not earlier, suggests a role in later-stage responses, potentially associated with attempts to limit tissue damage or regulate inflammation. Given the temporal association with increased mortality, it is plausible that A2M contributes to survival pathways under infectious stress, a hypothesis that remains unexplored in avian models and warrants further investigation. The present study not only identified a potential biomarker of inflammation in chickens but also highlighted a critical knowledge gap: whether A2M truly plays no role in the adaptive immune response in chickens, as it does in mammals, or whether its function in this species remains largely unexplored.

At the functional level, pathways, biological processes, and molecular functions showed slightly higher values in the infected groups. Although these differences did not reach statistical significance after correction for multiple testing, the credible intervals of the model estimates excluded zero in several comparisons, suggesting weak but consistent biological effects. As observed in the 24 h data set, PERMANOVA and ordination analyses revealed no statistically significant general differences between groups, supporting the idea of subtle, distributed proteomic modulation.

Despite the absence of statistically robust findings after multiple testing correction, some pathways exhibited relatively large fold changes and nominal p-values of <0.05, which should be interpreted as exploratory trends that may suggest biologically relevant responses. One notable example is the plasminogen activating cascade, which promotes the conversion of plasminogen into active plasmin. This pathway plays a central role not only in fibrinolysis and extracellular matrix remodeling but also in inflammation and tissue repair^31^ and may reflect early host responses to infection-induced tissue stress.

Similarly, the blood coagulation pathway was upregulated in infected embryos, likely reflecting the activation of the systemic inflammatory responses. Previous studies have shown that bacterial infection can trigger inflammation-driven coagulation cascades, emphasizing the strong link between coagulation and immune activation in chickens.^32^ In this context, increased coagulation activity may serve as an early and sensitive indicator of infection-induced inflammation.

In parallel, activation of the PPAR signaling pathway in infected embryos suggests an attempt to regulate inflammation and maintain metabolic balance. PPARs are nuclear receptors involved in lipid metabolism and immune modulation, and their activation can promote anti-inflammatory responses or facilitate pathogen persistence, depending on the context.^33^ In this study, PPAR signaling may reflect a compensatory mechanism aimed at modulating the host’s inflammatory state. These immunometabolic adaptations are further supported by upregulation of pathways involved in carbon and pyruvate metabolism, which may indicate increased energy demands in response to infection-induced stress. Such metabolic shifts are consistent with reprogramming events described in broiler chickens, where PPAR activation was associated with attenuation of inflammation and modulation of energy metabolism under challenging conditions^33^

Finally, the observed downregulation of mitophagy in infected embryos may compromise mitochondrial quality control during a critical period of embryonic development. Mitophagy is essential for eliminating damaged mitochondria and ensuring proper cell differentiation.^34,35^ Its suppression could therefore contribute to the developmental disturbances already observed in our data set. Similar findings have been reported in embryos exposed to environmental toxins, where impaired mitophagy led to oxidative damage and embryotoxicity,^35^ supporting a broader role for mitochondrial stress in mediating infection-related developmental impairment.

Although few proteins and pathways reached statistical significance after FDR correction, the proteomic profile at 48 h postinfection suggested a more established systemic inflammatory response compared to 24 h. This latter time point was characterized by increased abundance of proteins and pathways associated with coagulation, plasminogen activation, and metabolic regulation, including α-2-macroglobulin-like protein 1, plasminogen, blood coagulation, and the plasminogen activation cascade. In addition, some broader biological categories, including homeostatic, metabolic, and stimulus-response-related processes, showed positive logFC values at both time points, suggesting persistent physiological modulation following infection. In contrast, the 24 h profile appeared to reflect a more discrete and less organized biological response, suggesting a temporal progression of the host response following Salmonella Pullorum infection.

The use of CE as a model system, particularly before 12 DED, introduces several analytical challenges. Low protein levels, high biological variability, limited detection, and the lack of functional information for *Gallus gallus* make it difficult for conventional analyses to detect clear differences among groups. These limitations were addressed using Bayesian hierarchical models, including Tobit regression to handle censoring and mixed-effect models to account for variability across proteins and functional terms. These models proved effective at capturing nuanced, low-intensity signals associated with infection, providing a more interpretable representation of the host response under these constrained experimental conditions. However, the absence of significant PERMANOVA separation and the lack of FDR-significant changes for most proteins indicate that biological variability exceeded the detectable treatment effect at the proteome-wide level.

To our knowledge, this is the first study to characterize proteomic changes in the blood of CE-infected individuals prior to 12 DED. Moreover, this is the first report to associate A2M with an endogenous immune response in the embryonic or even adult bloodstream of *Gallus gallus*. While previous studies have documented the presence of this protein in egg white deposited during oogenesis, no prior work has demonstrated its differential expression in the embryonic liver or circulation in response to infection.

The metabolomic analysis revealed that NC and PC treatments induce general, time-dependent shifts in the metabolic composition, as evidenced by multivariate analyses. Although separation between experimental groups was already detectable at 24 h postinfection, a more pronounced divergence was observed at 48 h postinfection, indicating progressive metabolic remodeling following incubation. Importantly, these findings are methodologically consistent with the multivariate proteomic analyses, addressing potential concerns regarding the analytical coherence across omics layers.

Analysis of individual metabolites revealed specific changes underlying the observed group separation. At 24 h postinfection, α-CEHC and α-tocopheronolactone were increased in PC relative to NC, whereas several oxidized fatty acids, including 2R-hydroxylauric acid and 16-hydroxy-10-oxohexadecanoic acid, were reduced. These early alterations suggest a concurrent activation of antioxidant pathways and selective modulation of lipid-derived mediators even at 24 h postinfection.^36,37^

By 48 h postinfection, further divergence in metabolic profiles was apparent. Notably, phenylacetaldehyde showed a pronounced increase in PC embryos, along with sustained elevations in α-CEHC and α-tocopheronolactone. In contrast, several oxidized fatty acids, including 16-hydroxy-10-oxohexadecanoic acid and (−)-12-hydroxy-9,10-dihydrojasmonic acid, remained reduced.

Phenylacetaldehyde is a key aldehyde in the phenylalanine metabolic pathway and is readily oxidized to phenylacetic acid, which is primarily excreted in urine in its conjugated form.^38^ Although not commonly discussed in the context of inflammation, it has been detected in untargeted plasma metabolomics data sets from vertebrate models, including bovine plasma.^39^ Previous studies have reported elevated concentrations of phenylalanine-derived metabolites in children experiencing acute asthma exacerbations compared with healthy controls.^40,41^ The mechanisms linking inflammation and phenylalanine metabolism remain incompletely understood, but evidence suggests shared regulatory pathways: Inflammation can impair phenylalanine hydroxylase (PAH) activity, resulting in phenylalanine accumulation and diversion toward alternative catabolic routes, potentially increasing production of aldehyde intermediates such as phenylacetaldehyde.^42^

Among the metabolites contributing to group separation, α-CEHC represents the terminal product of enzymatic α-tocopherol degradation and is widely recognized as a functional marker of vitamin E metabolism under conditions of increased oxidative demand.^36,37^ In parallel, α-tocopheronolactone showed a tendency toward higher levels in infected embryos (*p* = 0.06), further supporting activation of vitamin E-related metabolic pathways. From the 10DED onward, increased oxygen consumption and metabolic activity lead to enhanced generation of reactive oxygen species (ROS), necessitating active antioxidant systems to maintain redox homeostasis during embryonic development.^43^ In this context, the increased levels of α-tocopherol and its metabolites in infected embryos are consistent with infection-associated oxidative challenge and suggest activation of vitamin E-dependent antioxidant pathways. These findings indicate that even at early developmental stages, chicken embryos can dynamically modulate redox defenses in response to inflammatory stress.^44^

Beyond antioxidant metabolism, alterations were also observed in the oxidized fatty acid derivatives. 16-Hydroxy-10-oxohexadecanoic acid, a long-chain fatty acid associated with lipid peroxidation and metabolism, was reduced in infected embryos.^45^ Hydroxy- and oxo-fatty acids, including 2R-hydroxylauric acid and 16-hydroxy-10-oxohexadecanoic acid, are functionally classified as oxylipins, a group of bioactive lipid mediators generated through fatty acid oxidation. Oxylipins encompass both pro- and anti-inflammatory molecules, and their production varies with the stages of inflammatory initiation, propagation, and resolution.^46^ Studies of systemic inflammation have demonstrated that oxylipin synthesis is highly dynamic and temporally regulated, with distinct mediator classes activated at different phases of the inflammatory response.^47^

Therefore, the observed alterations in oxidized fatty acid derivatives likely reflect dynamic remodeling of lipid mediator pathways rather than a simple attenuation of inflammation. Reduced levels of specific oxidized fatty acids in infected embryos may indicate their consumption or metabolic conversion as inflammation progresses. This dynamic regulation of lipid mediators may also be linked to the sustained increase in α-tocopherol metabolites observed at 24 and 48 h postinfection, suggesting activation of antioxidant mechanisms to control lipid peroxidation during *Salmonella*-induced inflammation.

This study demonstrates that the CE before 12 DED is a sensitive and tractable model for studying infection and inflammation. Proteomic analyses suggested modest and temporally structured shifts in protein abundance, although most proteome-wide differences were not statistically significant after multiple testing correction. Metabolomic analyses complemented these findings by showing time-dependent shifts in metabolic profiles, including increases in α-CEHC and α-tocopheronolactone, reflecting activation of antioxidant pathways, and dynamic changes in oxidized fatty acids indicative of lipid mediator remodeling. Together, these findings suggest subtle alterations in immune, antioxidant, and lipid-related pathways during early infection. The integration of proteomic and metabolomic evidence not only reinforces the sensitivity and mechanistic relevance of the CE model but also supports its use for identifying biomarkers of systemic inflammation and exploring early avian immune responses.

## Supplementary Material



## Data Availability

The mass spectrometry proteomics data have been deposited to the ProteomeXchange Consortium via the PRIDE partner repository (http://www.ebi.ac.uk/pride) with the data set identifier PXD072137.

## Notes

Sample allocation for proteomic and metabolomic analyses (Table S1) (PDF)

## References

[1] Domínguez-Oliva A., Hernández-Ávalos I., Martínez-Burnes J., Olmos-Hernández A., Verduzco-Mendoza A., Mota-Rojas D. (2023). The Importance of Animal Models in Biomedical Research:
Current Insights and Applications. Animals.

[2] Zosen D., Hadera M. G., Lumor J. S., Andersen J. M., Paulsen R. E. (2021). Chicken
Embryo as Animal Model to Study Drug Distribution to the Developing
Brain. J. Pharmacol. Toxicol. Methods.

[3] Garcia P., Wang Y., Viallet J., Macek Jilkova Z. (2021). The Chicken
Embryo Model: A Novel and Relevant Model for Immune-Based Studies. Front. Immunol..

[4] Kollmansperger S., Anders M., Werner J., Saller A. M., Weiss L., Süß S. C., Reiser J., Schneider G., Schusser B., Baumgartner C., Fenzl T. (2023). Nociception in Chicken
Embryos, Part II: Embryonal Development of Electroencephalic Neuronal
Activity In Ovo as a Prerequisite for Nociception. Animals.

[5] Banfield, B. W. ; Yap, G. S. ; Knapp, A. C. ; Enquist, L. W. A Chicken Embryo Eye Model for the Analysis of Alphaherpesvirus Neuronal Spread and Virulence 1998 72 4580 4588 10.1128/JVI.72.6.4580-4588.1998. PMC109971 9573221

[6] Ribeiro L., Schlemper A., Silva M., Fonseca B. (2022). Chicken Embryo: A Useful
Animal Model for Drug Testing?. Eur. Rev. Med.
Pharmacol Sci..

[7] Horvatić A., Guillemin N., Kaab H., McKeegan D., O’Reilly E., Bain M., Kuleš J., Eckersall P. D. (2019). Quantitative
Proteomics Using Tandem Mass Tags in Relation to the Acute Phase Protein
Response in Chicken Challenged with *Escherichia Coli* Lipopolysaccharide Endotoxin. J. Proteomics.

[8] Abudurexiti M., Abuduhalike R., Naman T., Wupuer N., Duan D., Keranmu M., Mahemuti A. (2024). Integrated Proteomic and Metabolomic
Profiling Reveals Novel Insights on the Inflammation and Immune Response
in HFpEF. BMC Genomics.

[9] de
Souza J. B., Sommerfeld S., Almeida-Souza H. O., Vaz E. R., Bastos L. M., Santos F., de A. A., Rodrigues A. C., Medeiros-Ronchi A. A., Goulart L. R., Fonseca B. B. (2024). A New Standardization
for the Use of Chicken Embryo: Selection of Target from the Phage
Display Library and Infection. Appl. Microbiol.
Biotechnol..

[10] Mi H., Ebert D., Muruganujan A., Mills C., Albou L.-P., Mushayamaha T., Thomas P. D. (2021). PANTHER Version 16: A Revised Family
Classification, Tree-Based Classification Tool, Enhancer Regions and
Extensive API. Nucleic Acids Res..

[11] Sollis E., Mosaku A., Abid A., Buniello A., Cerezo M., Gil L., Groza T., Güneş O., Hall P., Hayhurst J., Ibrahim A., Ji Y., John S., Lewis E., MacArthur J. A. L., McMahon A., Osumi-Sutherland D., Panoutsopoulou K., Pendlington Z., Ramachandran S., Stefancsik R., Stewart J., Whetzel P., Wilson R., Hindorff L., Cunningham F., Lambert S. A., Inouye M., Parkinson H., Harris L. W. (2023). The NHGRI-EBI GWAS Catalog: Knowledgebase
and Deposition Resource. Nucleic Acids Res..

[12] Koopmans F., Cornelisse L. N., Heskes T., Dijkstra T. M. H. (2014). Empirical Bayesian
Random Censoring Threshold Model Improves Detection of Differentially
Abundant Proteins. J. Proteome Res..

[13] Li Q., Li Y., Xia J., Wang X., Yin K., Hu Y., Yin C., Liu Z., Jiao X. (2019). Virulence of Salmonella Entericaserovar
Pullorum Isolates Compared Using Cell-Based and Chicken Embryo Infection
Models. Poult. Sci..

[14] O’Connell J. D., Paulo J. A., O’Brien J. J., Gygi S. P. (2018). Proteome-Wide Evaluation
of Two Common Protein Quantification Methods. J. Proteome Res..

[15] Rubin M. (2024). Inconsistent
Multiple Testing Corrections: The Fallacy of Using Family-Based Error
Rates to Make Inferences about Individual Hypotheses. Methods Psychol..

[16] Diz A. P., Carvajal-Rodríguez A., Skibinski D. O. F. (2011). Multiple
Hypothesis Testing in Proteomics: A Strategy for Experimental Work. Mol. Cell. Proteomics.

[17] Reed E. C., Silva V. A., Giebel K. R., Natour T., Lauten T. H., Jojo C. N., Schlieker A. E., Case A. J. (2025). Hemoglobin Alpha
Is a Redox-Sensitive Mitochondrial-Related Protein in T-Lymphocytes. Free Radic. Biol. Med..

[18] Carbon S., Douglass E., Good B. M., Unni D. R., Harris N. L., Mungall C. J., Basu S., Chisholm R. L., Dodson R. J., Hartline E., Fey P., Thomas P. D., Albou L.-P., Ebert D., Kesling M. J., Mi H., Muruganujan A., Huang X., Mushayahama T., LaBonte S. A., Siegele D. A., Antonazzo G., Attrill H., Brown N. H., Garapati P., Marygold S. J., Trovisco V., dos Santos G., Falls K., Tabone C., Zhou P., Goodman J. L., Strelets V. B., Thurmond J., Garmiri P., Ishtiaq R., Rodríguez-López M., Acencio M. L., Kuiper M., Lægreid A., Logie C., Lovering R. C., Kramarz B., Saverimuttu S. C. C., Pinheiro S. M., Gunn H., Su R., Thurlow K. E., Chibucos M., Giglio M., Nadendla S., Munro J., Jackson R., Duesbury M. J., Del-Toro N., Meldal B. H. M., Paneerselvam K., Perfetto L., Porras P., Orchard S., Shrivastava A., Chang H.-Y., Finn R. D., Mitchell A. L., Rawlings N. D., Richardson L., Sangrador-Vegas A., Blake J. A., Christie K. R., Dolan M. E., Drabkin H. J., Hill D. P., Ni L., Sitnikov D. M., Harris M. A., Oliver S. G., Rutherford K., Wood V., Hayles J., Bähler J., Bolton E. R., De Pons J. L., Dwinell M. R., Hayman G. T., Kaldunski M. L., Kwitek A. E., Laulederkind S. J. F., Plasterer C., Tutaj M. A., Vedi M., Wang S.-J., D’Eustachio P., Matthews L., Balhoff J. P., Aleksander S. A., Alexander M. J., Cherry J. M., Engel S. R., Gondwe F., Karra K., Miyasato S. R., Nash R. S., Simison M., Skrzypek M. S., Weng S., Wong E. D., Feuermann M., Gaudet P., Morgat A., Bakker E., Berardini T. Z., Reiser L., Subramaniam S., Huala E., Arighi C. N., Auchincloss A., Axelsen K., Argoud-Puy G., Bateman A., Blatter M.-C., Boutet E., Bowler E., Breuza L., Bridge A., Britto R., Bye-A-Jee H., Casas C. C., Coudert E., Denny P., Estreicher A., Famiglietti M. L., Georghiou G., Gos A., Gruaz-Gumowski N., Hatton-Ellis E., Hulo C., Ignatchenko A., Jungo F., Laiho K., Le Mercier P., Lieberherr D., Lock A., Lussi Y., MacDougall A., Magrane M., Martin M. J., Masson P., Natale D. A., Hyka-Nouspikel N., Orchard S., Pedruzzi I., Pourcel L., Poux S., Pundir S., Rivoire C., Speretta E., Sundaram S., Tyagi N., Warner K., Zaru R., Wu C. H., Diehl A. D., Chan J. N., Grove C., Lee R. Y. N., Muller H.-M., Raciti D., Van Auken K., Sternberg P. W., Berriman M., Paulini M., Howe K., Gao S., Wright A., Stein L., Howe D. G., Toro S., Westerfield M., Jaiswal P., Cooper L., Elser J. (2021). The Gene Ontology
Resource: Enriching a GOld Mine. Nucleic Acids
Res..

[19] Liao L., Yao Z., Kong J., Zhang X., Li H., Chen W., Xie Q. (2022). Transcriptomic Analysis Reveals the Dynamic Changes of Transcription
Factors during Early Development of Chicken Embryo. BMC Genomics.

[20] Vandooren J., Itoh Y. (2021). Alpha-2-Macroglobulin in Inflammation,
Immunity and Infections. Front. Immunol..

[21] Bergwik J., Kristiansson A., Allhorn M., Gram M., Åkerström B. (2021). Structure,
Functions, and Physiological Roles of the Lipocalin Α1-Microglobulin
(A1M). Front. Physiol..

[22] Sottrup-Jensen L. (1989). α-Macroglobulins:
Structure, Shape, and Mechanism of Proteinase Complex Formation. J. Biol. Chem..

[23] Lagrange J., Lecompte T., Knopp T., Lacolley P., Regnault V. (2022). Alpha-2-macroglobulin
in Hemostasis and Thrombosis: An Underestimated Old Double-edged Sword. J. Thromb. Haemostasis.

[24] Rehman A. A., Ahsan H., Khan F. H. (2013). Alpha-2-macroglobulin:
A Physiological
Guardian. J. Cell. Physiol..

[25] Wang X., Xu G., Yang N., Yan Y., Wu G., Sun C. (2019). Differential
Proteomic Analysis Revealed Crucial Egg White Proteins for Hatchability
of Chickens. Poult. Sci..

[26] Lim W., Jeong W., Kim J.-H., Lee J.-Y., Kim J., Bazer F. W., Han J. Y., Song G. (2011). Differential Expression
of Alpha 2 Macroglobulin in Response to Dietylstilbestrol and in Ovarian
Carcinomas in Chickens. Reprod. Biol. Endocrinol..

[27] Pathirana A., Diao M., Huang S., Zuo L., Liang Y. (2016). Alpha 2 Macroglobulin
Is a Maternally-Derived Immune Factor in Amphioxus Embryos: New Evidence
for Defense Roles of Maternal Immune Components in Invertebrate Chordate. Fish Shellfish Immunol..

[28] Shen N., Li C., Yang S., Ma Y., Wang H.-L. (2023). Liver Proteomics
Analysis Reveals the Differentiation of Lipid Mechanism and Antioxidant
Enzyme Activity during Chicken Embryonic Development. Int. J. Biol. Macromol..

[29] Yang S., Wei Z., Wu J., Sun M., Ma Y., Liu G. (2021). Proteomic
Analysis of Liver Tissues in Chicken Embryo at Day 16 and Day 20 Reveals
Antioxidant Mechanisms. J. Proteomics.

[30] Peng M., Li S., He Q., Zhao J., Li L., Ma H. (2018). Proteomics
Reveals Changes in Hepatic Proteins during Chicken Embryonic Development:
An Alternative Model to Study Human Obesity. BMC Genomics.

[31] Syrovets T., Lunov O., Simmet T. (2012). Plasmin as a Proinflammatory
Cell
Activator. J. Leukoc. Biol..

[32] Li Z., Qi Z., Wang X., Lu L., Wang H., He Z., Chen Z., Shao Y., Tu J., Song X. (2023). Avian Pathogenic *Escherichia Coli* Infection Causes Infiltration of
Heterophilic Granulocytes of Chick Tracheal by the Complement and
Coagulation Cascades Pathway. BMC Vet. Res..

[33] Li Y., Li J., Liu X., Cheng Z., Gao N., Kang J., Wang X. (2025). Polygonatum
Sibiricum Polysaccharides Alleviate LPS-Induced Liver
Injury in Chicks by Suppressing Inflammation and Oxidative Stress
Through the PPAR Signaling Pathway. Antioxidants.

[34] Chen D., Ran D., Wang C., Liu Y., Ma Y., Song R., Gao Y., Liu Z. (2021). Role of Mitochondrial Dysfunction and PINK1/Parkin-Mediated
Mitophagy in Cd-Induced Hepatic Lipid Accumulation in Chicken Embryos. Life Sci..

[35] Liu H., Lin H., Xu T., Shi X., Yao Y., Khoso P. A., Jiang Z., Xu S. (2023). New Insights into Brain
Injury in
Chickens Induced by Bisphenol A and Selenium Deficiency—Mitochondrial
Reactive Oxygen Species and Mitophagy-Apoptosis Crosstalk Homeostasis. Sci. Total Environ..

[36] Varga Z., Kosaras E., Komodi E., Katko M., Karpati I., Balla J., Paragh G., Aisa M. C., Galli F. (2008). Effects of
Tocopherols and 2,2′-Carboxyethyl Hydroxychromans on Phorbol-Ester-Stimulated
Neutrophils. J. Nutr. Biochem..

[37] Radosavac D., Graf P., Polidori M. C., Sies H., Stahl W. (2002). Tocopherol
Metabolites 2, 5, 7, 8-Tetramethyl-2-(2’-Carboxyethyl)-6-Hydroxychroman
(α-CEHC) and 2, 7, 8- Trimethyl-2-(2’-Carboxyethyl)-6-Hydroxychroman
(γ-CEHC) in Human Serum after a Single Dose of Natural Vitamin
E. Eur. J. Nutr..

[38] Human Metabolome Database. “HMDB0006236: Phenylacetaldehyde.” HMDB. https://www.hmdb.ca/metabolites/HMDB0006236. (accessed February 09, 2026).

[39] Xiang X., Chang H., Khan S., Khan I. M., Zhu F., Yang L., Duan G., Li J., Nassar N., Dai F., Mao H. (2025). Comparative Analysis of Milk Production, Composition,
and Plasma Metabolomics across Various Dairy Goat Breeds in Yunnan
Province, China. Front. Vet. Sci..

[40] Li J., Li X., Liu X., Wang X., Li J., Lin K., Sun S., Yue H., Dai Y. (2022). Untargeted Metabolomic
Study of Acute
Exacerbation of Pediatric Asthma via HPLC-Q-Orbitrap-MS. J. Pharm. Biomed. Anal..

[41] Cottrill K. A., Chandler J. D., Kobara S., Stephenson S. T., Mohammad A. F., Tidwell M., Mason C., Van Dresser M., Patrignani J., Kamaleswaran R., Fitzpatrick A. M., Grunwell J. R. (2023). Metabolomics Identifies Disturbances in Arginine, Phenylalanine,
and Glycine Metabolism as Differentiating Features of Exacerbating
Atopic Asthma in Children. J. Allergy Clin.
Immunol.: Global.

[42] Strasser, B. ; Sperner-Unterweger, B. ; Fuchs, D. ; Gostner, J. M. . Mechanisms of Inflammation-Associated Depression: Immune Influences on Tryptophan and Phenylalanine Metabolisms; 2017 95–115. 10.1007/7854_2016_23. 27278641

[43] Yigit A. A., Panda A. K., Cherian G. (2014). The Avian Embryo and Its Antioxidant
Defence System. Worlds Poult. Sci. J..

[44] Surai P. F., Fisinin V. I., Karadas F. (2016). Antioxidant Systems in Chick Embryo
Development. Part 1. Vitamin E, Carotenoids and Selenium. Anim. Nutr..

[45] Human Metabolome Database. “HMDB0041287:16-hydroxy-10-oxohexadecanoic acid.” HMDB. https://www.hmdb.ca/metabolites/HMDB0041287. (accessed February 09, 2026).

[46] Misheva M., Johnson J., McCullagh J. (2022). Role of Oxylipins in the Inflammatory-Related
Diseases NAFLD, Obesity, and Type 2 Diabetes. Metabolites.

[47] Myers M. N., Chirivi M., dos Santos Neto J. M., Parales-Girón J., Worden L. C., Lock A. L., Contreras G. A. (2025). Dynamics
of Oxylipin Biosynthesis in Systemic Inflammation: Insights from a
Large Animal Model of Endotoxemia. Front. Immunol..

