# Supplementary material for: Integrated Proteomics and Metabolomics Can Shed Light on Potential Biomarkers of Inflammation and Infection in Chicken Embryos as an Experimental Model
Source: J Proteome Res. 2026 Sep 1;25(10):5159–72. doi: 10.1021/acs.jproteome.6c00153 (PMC13640419; doi:10.1021/acs.jproteome.6c00153)
Supplement: Supplementary file 1 [file pr6c00153_si_001.pdf]

Supporting Information

Integrated Proteomics and Metabolomics Can Shed Light on Potential Biomarkers of Inflammation and Infection in Chicken Embryos as an Experimental Model

Simone Sommerfeld[*1], Hebreia Oliveira Almeida Souza[2], Mario Machado Martins[2], Bianca Uliana Picolo[2], Isabelle Ezequiel Pedrosa[1], Bianca de Jesus e Silva[1], Anna Luiza Filsner Dias Strack[1], Danielle Silva Vieira[1], Lara Reis Gomes[1], Murilo Vieira da Silva[1], Victor Aguiar de Souza Penha[3], Belchiolina Beatriz Fonseca[1,2]*

1. Graduate Program of Veterinary Science of Federal University of Uberlândia, Rua Ceará Bloco 2D, Campus Umuarama – Bairro Umuarama - Uberlândia - MG - CEP 38405-302;
2. Graduate Program of Genetics and Biochemistry of Federal University of Uberlândia Rua Acre Bloco 2E, Campus Umuarama – Bairro Umuarama - Uberlândia - MG - CEP 38400-902;
3. Department of Integrative Biology, Michigan State University, Natural Science Building, 288 Farm Lane, Rm. 203 East Lansing, MI 48824, USA.

* corresponding author: simone.sommerfeld@ufu.br

TABLE OF CONTENTS

Supplementary table S1……………………………………………………...S-2

Supplementary table S1. Sample allocation for proteomic and metabolomic analyses

**Timepoint**	**Group**	**Embryos available**	**Proteomics subset (n)**	**Total metabolomics samples (n)**
24 h	NC	20	10	20
24 h	PC	20	10	13
48 h	NC	20	10	16
48 h	PC	14	10	9

NC: negative control. PC: Positive control
